# The Performance of Different Artificial Intelligence Models in Predicting Breast Cancer among Individuals Having Type 2 Diabetes Mellitus

**DOI:** 10.3390/cancers11111751

**Published:** 2019-11-08

**Authors:** Meng-Hsuen Hsieh, Li-Min Sun, Cheng-Li Lin, Meng-Ju Hsieh, Chung Y. Hsu, Chia-Hung Kao

**Affiliations:** 1 Department of Electrical Engineering and Computer Sciences, University of California, Berkeley, CA 94720, USA; emersonhsieh@berkeley.edu; 2 Department of Radiation Oncology, Zuoying Branch of Kaohsiung Armed Forces General Hospital, Kaohsiung 813, Taiwan; limin.sun@yahoo.com; 3 Institute of Medical Science and Technology, National Sun Yat-sen University, Kaohsiung 804, Taiwan; 4 Management Office for Health Data, China Medical University Hospital, Taichung 404, Taiwan; orangechengli@gmail.com; 5 College of Medicine, China Medical University, Taichung 404, Taiwan; 6 Department of Medicine, Poznan University of Medical Sciences, 60965 Poznan, Poland; 76519@student.ump.edu.pl; 7 Graduate Institute of Biomedical Sciences, China Medical University, Taichung 404, Taiwan; hsucy63141@gmail.com; 8 Department of Nuclear Medicine and PET Center, China Medical University Hospital, Taichung 404, Taiwan; 9 Department of Bioinformatics and Medical Engineering, Asia University, Taichung 404, Taiwan

**Keywords:** type II diabetes mellitus, breast cancer, artificial neural network, logistic regression, random forest

## Abstract

*Objective:* Early reports indicate that individuals with type 2 diabetes mellitus (T2DM) may have a greater incidence of breast malignancy than patients without T2DM. The aim of this study was to investigate the effectiveness of three different models for predicting risk of breast cancer in patients with T2DM of different characteristics. *Study design and methodology:* From 2000 to 2012, data on 636,111 newly diagnosed female T2DM patients were available in the Taiwan’s National Health Insurance Research Database. By applying their data, a risk prediction model of breast cancer in patients with T2DM was created. We also collected data on potential predictors of breast cancer so that adjustments for their effect could be made in the analysis. Synthetic Minority Oversampling Technology (SMOTE) was utilized to increase data for small population samples. Each datum was randomly assigned based on a ratio of about 39:1 into the training and test sets. Logistic Regression (LR), Artificial Neural Network (ANN) and Random Forest (RF) models were determined using recall, accuracy, F_1_ score and area under the receiver operating characteristic curve (AUC). *Results:* The AUC of the LR (0.834), ANN (0.865), and RF (0.959) models were found. The largest AUC among the three models was seen in the RF model. *Conclusions:* Although the LR, ANN, and RF models all showed high accuracy predicting the risk of breast cancer in Taiwanese with T2DM, the RF model performed best.

## 1. Introduction

Globally, diabetes mellitus (DM) accounts for a large proportion of the burden of chronic diseases. The World Health Organization’s global report indicates a gradual increase of DM in the last 30 years (increasing from 4.7% in 1980 to 8.5% in 2014) [1], which is becoming a major public health burden [2]. Among all DM types, type 2 DM (T2DM) accounts for the majority (90–95%) of cases. The standardized DM incidence rate in Taiwan reflects the global trend, with a near constancy noted over the years (0.805% in 2000 and 0.823% in 2008). By contrast, DM prevalence in Taiwan has steadily increased (3.34% in 2000 and 5.22% in 2008 in women; 3.01% in 2000 and 5.24% in 2008 in men), suggesting a possibility of relative success in DM treatment leading to lowering of death rates among affected persons [3].

With the increasing life-expectancy of individuals with DM, the development of subsequent DM-related complications in these patients is gaining public attention. DM might be a risk factor for several individual cancers [4,5,6,7,8,9,10]; specifically, some epidemiological studies have indicated that it can increase breast cancer risk [11,12,13,14,15,16,17].

Breast cancer, a leading malignancy in women globally, accounted for approximately one in four newly diagnosed female cancer cases worldwide in 2018 [18]. Since 1996, it is the most common cancer found in female Taiwanese. According to the National Cancer Registry, the age-adjusted incidence rate of breast malignancy sharply rose from 51.94/100,000 person-years in 2006 to 73.57/100,000 person-years in 2015 [19]. Furthermore, breast cancer in Taiwan is remarkable, as affected women tend to present at a lower age (45–49 years) compared with Caucasian Americans [20]; this may affect national productivity contributed by the active labor force from this age group. In Taiwan, through early detection and innovative treatment of breast cancer, we may be able to monitor the fluctuations in breast cancer incidence and decrease mortality rates in the near future [21]. Awareness regarding risk factors and efficient screening procedures could be a first step toward achieving this goal. 

Thus, in this study, we compared the performance of the logistic regression (LR), artificial neural network (ANN), and random forest (RF) models for the prediction of cancer of breast in Taiwanese women having T2DM with different parameters associated with T2DM.

## 2. Methods

### 2.1. Data Source

Since 1995, the government of Taiwan has had a National Health Insurance (NHI) scheme which has achieved 99% coverage of the population. This study used data retrieved from the NHI’s Longitudinal Cohort of Diabetes Patients, which consisted 1,700,000 individuals registered as new cases of T2DM based on the International Classification of Diseases, 9th Revision (ICD-9-CM, code 250x0 and code 250x2) and randomly selected patients from the NHI program. 

### 2.2. Data Availability Statement

The study utilized datasets available with the Taiwan Ministry of Health and Welfare (MOHW). Access to the datasets to researchers is granted after due application and approval by the MOHW. The MOHW can be contacted for access to the datasets through email (stcarolwu@mohw.gov.tw), at the Taiwan MHOW office address (No. 488, Sec. 6, Zhongxiao E. Rd., Nangang Dist., Taipei City 115, Taiwan (R.O.C.)) or by phone (+886-2-8590-6848).

### 2.3. Ethics Statement

All personal identification information was encrypted and only anonymized datasets were available to the researchers for analysis. The Institutional Review Board (IRB) of China Medical University (CMUH-104-REC2-115-CR3) reviewed the study protocol, approved it and affirmed that this study fulfilled the condition for exemption. The requirement of patient consent was also specifically waived as well.

### 2.4. Sampled Participants

Female patients whose records indicated a diagnosis of T2DM two or more times within one year during 2000–2012 were included. The date of first T2DM diagnosis was considered the index date. Female patients whose records indicated a diagnosis of breast cancer (ICD-9-CM code 174) before this date and were <20 years old were excluded. Background variables comprised age, urbanization level, and occupation. Comorbid conditions considered at baseline include: hyperlipidemia, high blood pressure, cerebrovascular accident, obesity, benign breast condition, congestive cardiac failure, chronic obstructive pulmonary disease (COPD), asthma, coronary artery disease (CAD), smoking, alcohol-related illness, and chronic kidney disease (CKD). Seven complication categories of the adapted Diabetes Complication Severity Index (aDCSI) were included: nephropathy, retinopathy, neuropathy, cardiovascular complications, cerebrovascular complications, peripheral vascular disease, and metabolic complications. Medications, such as statins, aspirin, estrogen, insulin, sulfonylureas, thiazolidinedione (TZD), and metformin, as well as other medications for treating patients with diabetes were considered if there were some correlational relationship between them and the development of breast cancer.

### 2.5. Training Set

The original raw data included gender, age, occupation, urbanization, and comorbidity. The urbanization and occupation levels of each subject were one-hot encoded, creating eight additional features in the processed data. All features were categorical except subject age, which was unity-based normalized and standardized on the basis of the training set. The processed data included 37 features. Mean imputation was used for subjects with missing values.

Data in the negative case outnumbered those in the positive case by a ratio of approximately 86:1. After data cleaning and feature processing, the Synthetic Minority Oversampling Technique (SMOTE) was applied to balance the positive and negative classes. The SMOTE created new minority data by interpolation within the available minority data via bootstrap sampling and data generation via the *k*-nearest neighbors algorithm [22]. SMOTE has been applied in machine learning applications in healthcare. Tun et al. and Alghamdi et al. used SMOTE to generate synthetic observations from datasets for bladder cancer prediction [23] and diabetes mellitus prediction [24], respectively. The K parameter, which determines the number of closest neighbors considered with each SMOTE iteration, was set to 5. To achieve an approximate balance between the positive and negative classes, 86 new data for the negative class were created for each datum of the positive class. Subsequently, random allocation was used to assign the data to the training and testing sets at a ratio of about 39:1. 

### 2.6. Algorithm Training and Evaluation

The ANN model consists of an input, three hidden layers and an output layer of 37, 20, and 2 dimensions, respectively. The model used the Scaled Exponential Linear Unit activation function after each hidden layer [25] and the Softmax activation function for the output layer. Model training was done through cross-entropy loss and optimized with Adam [26]. Dropout regularization of 20% and 50% were applied after the input and hidden layers, respectively [27]. The LR model used L_2_ loss for regularization, the liblinear solver as the optimizer [28], and the one-vs.-rest scheme as the loss function. The model was trained for 100 iterations and had regularization strength of 1.0. The RF model was trained with 20 decision trees with maximum tree depth of 10. The quality of split was measured using Gini impurity. Each leaf had a minimum of one sample, and each split had a minimum of two samples. Models building was done in Python (v. 3.7.0), along with the Tensorflow library (version 1.12.0) for the ANN model [29] and the scikit-learn library (v. 0.20.1) for the LR and RF models [30].

The *k*-fold cross-validation accuracy (*k* = 10) was used during model selection and tuning. The test set was not used during model tuning and was used only for model evaluation after the entire model selection and training process. The final models were evaluated using the confusion matrix metrics of precision (positive predictive value), recall (sensitivity), F_1_ (harmonic mean between precision and recall), and area under receiver operating characteristic (ROC) curve (AUC). The ROC curves were constructed based on the prediction probabilities, and the AUCs were compared using the DeLong test [31].

### 2.7. Statistical Analyses

The differences in sociodemographic distributions, underlying diseases, diabetes complications, and medications between patients with and without breast cancer were compared using the Student’s *t*-test (for quantitative variables such as age and aDCSI score) and Chi-square test (for proportions).

Data management was carried out with SAS (v.9.4; SAS Institute, Cary, NC, USA). All two-tailed *p* values of <0.05 were considered statistically significant.

## 3. Results

### 3.1. Patient Demographic Features

Compared with patients without breast cancer, patients with breast cancer tended to be slightly younger (56.9 ± 10.7 vs. 58.4 ± 14.2 years), live in urbanized areas (66.1% vs. 58.6%), have white-collar jobs (49.4% vs. 44.8%), have benign breast condition, and have estrogen use (Table 1). After the follow-up period, subjects with and without breast cancer had a mean (standard deviation) aDCSI score of 2.27 (1.96) and 3.12 (2.33), respectively.

### 3.2. Evaluation of Prediction Models

Table 2 lists the evaluation metrics of the confusion matrix and the AUCs of all the prediction models. Table 3 lists the *k*-fold cross-validation accuracies (*k* = 10) of all the prediction models. The LR model had the highest *k*-fold cross validation accuracy; in all other metrics, the RF model demonstrated the best performance.

**Figure 1:**
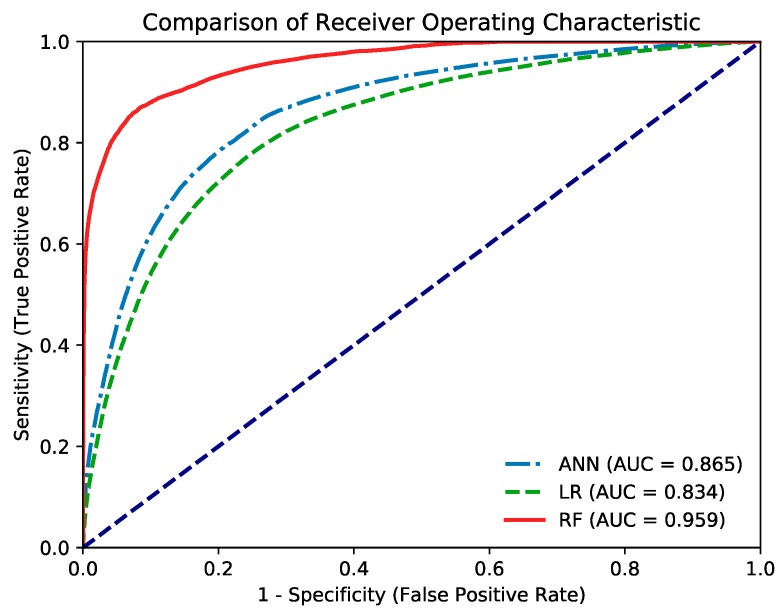
The receiver operating characteristic curve of the artificial neural network (ANN), logistic regression (LR), and random forest (RF) models in predicting breast cancer.

Figure 1 displays the ROC curves for the three models. The AUCs of the LR (0.834), ANN (0.865), and RF (0.959) models were determined. Although the RF model performed better than the ANN model in the training set and across all data, both models demonstrated similar performance in the test set. A significant difference was noted between the AUCs of all three models (*p* < 0.0001). Furthermore, the AUC of the RF model was significantly larger than that of the other two models.

## 4. Discussion

By using three models, we evaluated the possible increase in the risk of breast malignancy in Taiwanese female having T2DM with different features based on a national population-based database. The AUCs of all three models were all significantly larger than the 0.5. Although the LR model achieved the highest *k*-fold cross-validation accuracy among the three models, the RF model displayed the highest precision, recall, and F_1_, and the largest AUC. Furthermore, the AUC of the RF model was significantly larger than that of the other two models. 

T2DM may be associated with a high risk of certain cancers [4,5,6,7,8,9,10]. Cancer cells obtain bioenergy by consuming more glucose than normal cells do and thus achieve “out-of-control” growth [32]. From an organismal perspective, Ye et al. suggested that cancer cell populations can be considered similar to parasites which compete with the host for some essential systemic resources, such as glucose [9]. Several studies focusing on the relationship between breast cancer and T2DM have argued that T2DM predisposes women to having a greater risk of developing breast cancer than is the general population [11,12,13,14,15,16,17]. Despite T2DM and cancer sharing some risk factors (e.g., obesity, aging, high-fat diet, and insufficient physical exercise) [7], several biologically plausible mechanisms might account for why T2DM may be a predictor of breast cancer. First, insulin resistance-induced increase in insulin level can stimulate cell proliferation and promote mitogenic effects in breast tissue [33]. Moreover, breast cancer cells tend to have excessive expression of insulin receptors [34]. Second, T2DM is usually related to chronic low-grade inflammation, and long-term inflammation may trigger breast cancer development [35]. Third, medication used for T2DM may affect the relationship between T2DM and breast cancer. Medication that increases insulin levels may be associated with higher cancer risks [36,37]; by contrast, treatment with insulin sensitizers, including metformin, may reduce breast cancer risk [38,39]. In this study, we included medications that potentially affect this relationship to reduce the possible confounding effects.

Since the launch of the NHI program over two decades ago, universal health coverage has reached 99.6% of the total population in Taiwan through the government health care system. The NHI Research Database (NHIRD) is a reliable data resource for conducting national epidemiological studies in Taiwan. Several researchers have employed the NHIRD with traditional statistical methods to assess the association of DM and antidiabetic medications with breast cancer risk [15,16,37,39]. Liaw et al. applied a Cox proportional hazard regression model to verify the association between breast cancer risk with T2DM and found that women with T2DM had a substantially greater risk of breast cancer compared with those without DM [15]. Tseng used an LR method to assess the association between DM and the risk of breast malignancy and indicated that the significant association was considerably attenuated after adjustment for potential confounders (before adjustment: odds ratio 2.63; 95% confidence interval (95% C.I.) 2.31–2.98; after adjustment: odds ratio 1.81; 95% C.I. 1.59–2.06) [16]; however, women with DM may be less likely to receive mammography screening [40].

In addition to the LR method, we used two computational learning models (ANN and RF) to predict the risk of breast cancer in individuals with T2DM. LR is a classification algorithm and can be applied to determine the risk (odds) of a disease; its outcome can be binomial, ordinal, or multinomial, and the LR analysis is used to create a statistical model for a binary response data [41]. In medicine, LR is the most commonly used method for developing predictive models for dichotomous outcomes [42]. By contrast, ANNs are computational models inspired by biological neural networks; they are currently the commonest practiced models of artificial intelligence used for risk prediction and decision-making [42,43]. Furthermore, ANNs are suitable for NHIRD-based prediction of certain illnesses [44,45]. RF modeling is an ensemble learning method that performs a computationally extensive and robust data-mining and can accommodate large sets of proposed variables as inputs to identify factors associated with the outcomes of interest [46]. Several decision trees are developed at training time, and the output is the class that is the form of the classification or regression of the individual trees [47]. We used the *k*-fold cross-validation accuracy during the model selection process and found that the LR model achieved the highest *k*-fold validation accuracy among the three models. The final classification models were evaluated using the confusion matrix metrics of recall, precision, and F_1._ In addition, the ROC curve summarizes the model’s performance by evaluating the tradeoffs between false positive rate (1 − specificity) and true positive rate (sensitivity). The AUC is the performance metric for the ROC curve: the higher is the AUC, the higher is the prediction power of the model. The AUCs of the three models were all significantly higher than 0.5, suggesting a direct relationship between T2DM and the occurrence of breast cancer. Our analyses indicated that the RF model performed the best according to the precision, recall, F_1_, and AUC; the AUC of the RF model was significantly larger than that of the other models. While generalized linear models such as the LR model are not strictly more interpretable than the ANN model [48], the RF model performs better than both models for the dataset in this study and is an interpretable model.

This study is the first nationwide-based investigation known to the authors that used three prediction models to evaluate breast cancer risk among women with T2DM with different clinical and demographic characteristics associated with T2DM. As shown in Table 1, we included available potential risk factors and antidiabetic medications that may be related to breast cancer in the current algorithm to obtain an accurate prediction. However, some limitations need to be considered when interpreting the findings of this study. First, a detailed profile regarding lifestyle behaviors, such as alcohol consumption, smoking status, family history, body mass index, diet, and physical activity (related to T2DM, breast cancer or both), is lacking in the NHIRD, which may cause some confounding effects. To reduce these effects, we used the comorbidities as potential surrogates for some determinants, e.g., alcohol-related illness for alcohol, COPD and attendance of a smoking-cessation clinic for smoking, and obesity for body mass index. Second, inherent limitations of the NHIRD prevented us from obtaining histological patterns, grading, staging information, biochemical data, and molecular markers of breast cancer, thus impeding more comprehensive analyses. There was also a lack of image-based data; with such data, frameworks such as the one described by Dimitriou et al. for stage II colorectal cancer prognosis could be applied and the results could be directly compared [49]. Third, the analyses did not differentiate the importance of potential clinical and demographic predictors, which may raise the question regarding whether this approach can be practicable clinically. While the main purpose of this study was to explore the feasibility of using various machine learning models for predicting breast cancer among patients with breast cancer, an interpretation of a trained RF model for this application can be explored further in future studies. Fourth, the performance of different machine learning models, such as the support vector machine (SVM) used by Ferroni et al. to predict breast cancer, can be further studied for this application [50]. Fifth, the study did not use an external dataset for validation, and all validation was done with *k*-fold cross validation and a test set that was not used during the training process. Such validation can be an avenue for a future study.

## 5. Conclusions

T2DM with different features may be an independent risk factor for breast cancer in Taiwanese women. Moreover, among all models, the RF model was the most effective at predicting breast cancer. Because the median age at breast cancer diagnosis in Taiwan is relatively young, our study indicates that breast cancer surveillance policy may require modification to include T2DM patients in the earlier stages of breast cancer detection.

## Figures and Tables

**Table 1:** Baseline characteristics of T2DM patients with and without breast cancer.

Variable	Breast Cancer				
	No		Yes		*p* Value
	*N* = 628765		*N* = 7346		
	*n*	(%)	*n*	(%)	
Age group (year)					<0.001
≤49	171,724	27.3	1943	26.5	
50–64	251,750	40.0	3716	50.6	
65+	205,291	32.7	1687	23.0	
Mean (SD) (year) *	58.4	14.2	56.9	10.7	
Urbanization level #					<0.001
1 (highest)	183,283	29.2	2589	35.2	
2	185,090	29.4	2272	30.9	
3	100,217	15.9	1049	14.3	
4 (lowest)	160,175	25.5	1436	19.6	
Occupation					<0.001
White collar	281,372	44.8	3632	49.4	
Blue collar	294,699	46.9	3127	42.6	
Others ‡	52,694	8.38	587	7.99	
Underlying disease					
Hypertension	470,048	74.8	5236	71.3	<0.001
Hyperlipidemia	435,254	69.2	5046	69.7	0.33
Stroke	88,246	14.0	606	8.25	<0.001
Congestive heart failure	95,160	15.1	645	8.78	<0.001
Benign breast condition	111,647	17.8	4899	66.7	<0.001
Obesity	42,712	6.79	479	6.52	0.36
COPD	164,128	26.1	1619	22.0	<0.001
CAD	250,789	39.9	2574	35.0	<0.001
Asthma	138,917	22.1	1256	17.1	<0.001
Stop-smoking clinic	6107	0.97	28	0.38	<0.001
Alcohol-related illness	26,210	4.17	216	2.94	<0.001
CKD	188,584	30.0	1632	22.2	<0.001
Diabetes complication (components of the aDCSI)					
Retinopathy	127,829	20.3	1123	15.3	<0.001
Nephropathy	222,113	35.3	1925	26.2	<0.001
Neuropathy	212,414	33.8	2025	27.6	<0.001
Cerebrovascular	168,028	26.7	1257	17.1	<0.001
Cardiovascular	383,242	61.0	3906	53.2	<0.001
Peripheral vascular disease	179,865	28.6	1419	19.3	<0.001
Metabolic	25,411	4.04	149	2.03	<0.001
Mean aDCSI score (SD)					
Onset	1.62	1.68	1.29	1.46	<0.001
End of follow-up	3.12	2.33	2.27	1.96	<0.001
Medications					
Statin	349,906	55.7	3465	47.2	<0.001
Aspirin	30,561	4.86	176	2.40	<0.001
Estrogen	274,204	43.6	3416	46.5	<0.001
Insulin	191,580	30.5	1181	16.1	<0.001
Sulfonylureas	340,489	54.2	3698	50.3	<0.001
Metformin	389,319	61.9	3897	53.1	<0.001
TZD	101,370	16.1	815	11.1	<0.001
Other antidiabetic drugs	167,166	26.6	1414	19.3	<0.001

# Urbanization level was divided into four different categories according to the population of the residential areas; level 1 = “most urbanized” to level 4 = “least urbanized”. ‡ Other occupations, e.g., “retired”, “unemployed”, or “low income populations”. aDCSI, adapted Diabetes Complication Severity Index. Chi-square test, and * *t*-test comparing subjects with and without breast cancer.

**Table 2:** Metrics of the ANN, LR, and RF models.

Dataset	Model	F_1_	Precision	Recall	AUROC	AUROC SE	AUROC 95% CI
All (*n* = 1,267,867)	ANN	0.789	0.791	0.790	0.865	<0.001	0.864–0.866
	LR	0.763	0.765	0.763	0.834	<0.001	0.833–0.835
	RF	0.892	0.892	0.892	0.959	<0.001	0.959–0.960
Train (*n* = 1,236,170)	ANN	0.789	0.791	0.790	0.865	<0.001	0.864–0.866
	LR	0.763	0.765	0.763	0.834	<0.001	0.833–0.835
	RF	0.892	0.892	0.892	0.960	<0.001	0.959–0.960
Test (*n* = 31,697)	ANN	0.789	0.790	0.789	0.864	0.002	0.860–0.868
	LR	0.758	0.761	0.758	0.829	0.002	0.824–0.833
	RF	0.890	0.890	0.890	0.955	0.003	0.948–0.961

**Table 3:** The *k*-fold cross-validation accuracy (*k* = 10) of all three prediction models.

Model	ANN	LR	RF
*k*-fold accuracy	0.786	0.881	0.763
